# Diverse Genotypes of *Cronobacter* spp. Associated with Dairy Farm Systems in Jiangsu and Shandong Provinces in China

**DOI:** 10.3390/foods13060871

**Published:** 2024-03-13

**Authors:** Hui Liu, Xing Ji, Haichang Sun, Craig Billington, Xiang Hou, Abbas Soleimani-Delfan, Ran Wang, Heye Wang, Lili Zhang

**Affiliations:** 1 Key Laboratory of Food Quality and Safety of Jiangsu Province—State Key Laboratory Breeding Base, Institute of Food Safety and Nutrition, Jiangsu Academy of Agricultural Sciences, Nanjing 210014, Chinajixing@jaas.ac.cn (X.J.); asdelfan2003@gmail.com (A.S.-D.);; 2 College of Animal Science and Technology, Guangxi University, Nanning 530004, China; 3 College of Veterinary Medicine, Nanjing Agricultural University, Nanjing 210095, China; 4 Institute of Environmental Science and Research, 27 Creyke Road, Ilam, Christchurch 8041, New Zealand; craig.billington@esr.cri.nz; 5 International Phage Research Center, Jiangsu Academy of Agricultural Sciences, Nanjing 210014, China

**Keywords:** *Cronobacter* spp., whole-genome sequencing, antimicrobial susceptibility, phylogenetic analysis

## Abstract

*Cronobacter* spp. are the most concerning foodborne pathogen in infant formula milk powder. Currently, there are many reports on the prevalence of *Cronobacter* spp. in infant formula milk and its processing environment, but there are few studies on the prevalence of *Cronobacter* spp. on dairy farms. We have, therefore, undertaken this study to investigate and track genomic epidemiology of *Cronobacter* spp. isolates from Chinese dairy farms in the provinces of Jiangsu and Shandong. In this study, forty *Cronobacter* spp. strains, consisting of thirty *Cronobacter sakazakii*, eight *Cronobacter malonaticus*, and two *Cronobacter dublinensis*, were obtained from 1115 dairy farm samples (raw milk, silage, bedding, and feces), with a prevalence rate of 3.57%. These isolates were classified into 10 *Cronobacter* serotypes and 31 sequence types (STs), including three novel STs which were isolated for the first time. Notably, pathogenic *Cronobacter* STs 7, 8, 17, 60, and 64, which are associated with clinical infections, were observed. Antimicrobial susceptibility testing showed that all the *Cronobacter* spp. were highly resistant to cephalothin and fosfomycin, which was consistent with the antimicrobial genotype. All isolates carried core virulence genes related to adherence, invasion, endotoxin, immune evasion, secretion system, and regulation. Approximately half the isolates were also able to produce a strong biofilm. Twenty-one prophages and eight plasmids were detected, with the most common prophage being *Cronobacter*_ENT47670 and the most common plasmid being IncFIB (pCTU1). In addition, two isolates harbored the transmissible locus of stress tolerance (tLST) which confers high environmental persistence. Phylogenetic analysis showed strong clustering by species level and sequence types. Isolates from different sources or regions with a similar genomic background suggests the cross-contamination of *Cronobacter* spp. The presence of diverse genotypes of *Cronobacter* spp. in dairy farms in Jiangsu and Shandong provinces indicates that surveillance of *Cronobacter* spp. on dairy farms should be strengthened, to prevent and control transmission and ensure the quality and safety of raw dairy products.

## 1. Introduction

*Cronobacter* species are Gram-negative opportunistic pathogens that can cause clinical infections in people of all age groups, but especially in immunocompromised and underweight infants. Its occurrence can lead to infant septicemia, meningitis, and necrotizing enterocolitis with a mortality rate of up to 50%. This genus comprises seven species, including *C. sakazakii*, *C. malonaticus*, *C. turicensis*, *C. muytjensii*, *C. dublinensis*, *C. condimenti*, and *C. universalis* [1]. The primary pathogenic species found to cause clinical infections are *C. sakazakii* and *C. malonaticus* [2,3]. *Cronobacter* spp. are ubiquitous in the environment and have been isolated from foods and environmental samples, including cereals, milk powder, vegetables, fruits, plants, feces, and river water [4,5,6].

Antibiotics remain the most effective means of treating bacterial infections globally [7]. In recent years, public support for better antimicrobial stewardship has led to tighter regulations on antibiotic use globally, but there remain issues in controlling antimicrobial resistance in foodborne pathogens. Previous studies have reported that most *Cronobacter* spp. are sensitive to conventional antimicrobials, but the proportion of *Cronobacter* spp. exhibiting multiple-drug resistance is still increasing year by year [8,9]. Therefore, understanding the prevalence of *Cronobacter* and its antibiotic resistance in farming systems will help develop effective monitoring systems and contribute to improving public health and help control the spread of antibiotic resistance.

Understanding the genetic diversity of pathogens can contribute to accurate identification at the genus and species levels and reveal genetic relationships between strains. Several molecular typing methods have been implemented for the characterization of *Cronobacter* spp., including multilocus sequence typing (MLST) and pulsed-field gel electrophoresis (PFGE), over the past decade [10,11]. Whole genome sequencing (WGS) analysis has been progressively replacing these methods as it offers a much higher level of discrimination and greater information about pathogenic strains, such as antimicrobial resistance and virulence genes [12].

Many studies have shown that *Cronobacter* spp. has good environmental stress tolerance, associated with the presence of a transmissible locus of stress tolerance (tLST), previously termed the locus of heat resistance (LHR), which confers resistance to heat [13,14]. The tolerance to heat is not very high in many strains, but it is increased in strains possessing tLST. The tLST is composed of some heat shock-encoding genes, including those encoding the small heat shock protein sHSP20, heat resistance protein PsiE-GI, and heat resistance proteins YfdX1 and YfdX2, which can be mobilized by horizontal gene transfer in some *Enterobacteriaceae* [15]. In addition, biofilm formation also helps bacteria to resist various environmental stressors. Bacteria are capable of forming biofilms on the surface of materials, increasing the possibility of environmental persistence of this pathogen [16].

The occurrence of *Cronobacter* spp. in foods may be due to cross-contamination during manufacture and storage. *Cronobacter* spp. infections in infants are mainly linked to the consumption of contaminated powdered infant formula. A large amount of research has been focused on infant formula milk and its processing environment since *Cronobacter* spp. was first detected in powdered infant formula [17,18]. It has been shown that *Cronobacter* spp. can be disseminated by breast milk and in breast pumps [19,20]. However, risk assessments and investigations into the genetic characteristics of *Cronobacter* spp. in raw milk and the living environments of cows on the farm are rare. Hana Vojkovska et al. and Catherine Molloy et al. investigated the *Cronobacter* spp. isolated from foods of plant origin and from farm environmental samples [21,22]. To our knowledge, this is the first time that an analysis of *Cronobacter* spp. in raw milk and dairy farm environments in China has been reported.

Developing a better understanding of *Cronobacter* spp. on dairy farms is important to elucidate dissemination routes on farms and to help prevent food- and food animal-based transmission in the supply dairy chain. The objective of this study was to investigate the prevalence and distribution of *Cronobacter* spp. in Jiangsu and Shandong province dairy farms, using phenotyping and genotyping methods in order to understand the genetic relatedness of isolates and for the evaluation of virulence factors.

## 2. Materials and Methods

### 2.1. Sample Collection and Cronobacter spp. Identification

A total of 1115 unique samples, including raw milk (n = 710), silage (n = 100), bedding (n = 155), and cow feces (n = 150), were collected from commercial dairy farms located in Jiangsu and Shandong provinces in China from 2021 to 2023. Raw milk samples were randomly collected from healthy cows by mechanical milking, and environmental samples (silage, bedding, and cow feces) were collected from different areas of the cowshed. Samples were collected in sterile screwed bottles and sampled bags, quickly stored at 4 °C, and transported to the laboratory for bacteriological analysis. Details of the sampling, the number of milking cows at each dairy farm, and the collection time are given in Supplementary Table S1. The samples were diluted 10-fold in sterile buffered peptone water (BPW, Thermo Fisher Scientific Co., Ltd., Shanghai, China), and then 100 μL of diluted sample was plated onto chromogenic medium *Cronobacter* spp. agar (CHROMagar, Paris, France). Blue-green colonies on the media were identified as presumptive *Cronobacter* spp. and were confirmed by the species-specific *fusA* gene sequencing, as previously described [23], and VITEK 2 Compact Gram-negative identification card analysis (bioMérieux, Marcy-l’Étoile, France).

### 2.2. Whole-Genome Sequencing and Bioinformatics Analysis

All confirmed *Cronobacter* spp. isolates were subjected to whole-genome sequencing. Genomic DNA from each isolate was extracted using the Bacteria DNA Extraction Kit (Magen, Guangzhou, China) following the manufacturer’s protocols, then sequencing was completed using the Illumina HiSeq X-Ten System (Illumina Inc., San Diego, CA, USA). Sequence reads were assembled using SPAdes (http://cab.spbu.ru/software/spades/, accessed on 12 May 2023) and quality-filtered using Unicycler (version 0.4.8). The final identification of the *Cronobacter* spp. strains was confirmed with the Ribosomal Multilocus Sequence Typing (rMLST) (https://pubmlst.org/species-id, accessed on 27 May 2023) [6]. Multiple-locus sequence typing (MLST) of *Cronobacter* spp. was performed by uploading the whole-genome sequence to the PubMLST *Cronobacter* spp. (https://pubmlst.org/organisms/cronobacter-spp/, accessed on 27 May 2023). The presence of the serotype O region-specific *gnd* and *galF* genes was determined by analyzing WGS sequences with the Bacterial Isolate Genome Sequence Database (BIGSdb) tools in the PubMLST (http://pubmlst.org/, accessed on 27 May 2023). All known resistance and virulence genes were screened using the ResFinder and VirulenceFinder databases (>90% identity) [24]. Prophages were identified from the assembled chromosomes of isolates using the Prophage Hunter tool (https://pro-hunter.genomics.cn, accessed on 27 May 2023) [25]. Plasmids were detected by the online analysis tool PlasmidFinder (http://www.genomicepidemiology.org/, accessed on 2 July 2023) [26]. The tLST was analyzed from the assembled chromosomes of *Cronobacter* spp. isolates. The tLST sequences were run through the automatic annotation pipeline RAST (https://rast.nmpdr.org/, accessed on 11 July 2023), and a comparison of the genetic context was generated using BLASTn and further visualized using Easyfig (v2.2.2) [27]. The genome assemblies of isolates were deposited in GenBank and registered with the BioProject number PRJNA995030.

### 2.3. Antimicrobial Susceptibility Testing

Antimicrobial susceptibility testing was carried out using the broth microdilution method according to Clinical and Laboratory Standards Institute 2021 guidelines, including the following 10 antimicrobial agents: ampicillin, cephalothin, tetracycline, ciprofloxacin, gentamicin, clindamycin, sulfamethoxazole, meropenem, chloramphenicol, and fosfomycin. *Escherichia coli* ATCC 25922 was used as a quality control strain.

### 2.4. Biofilm Formation Assays

The ability of *Cronobacter* spp. to form biofilms was determined using a crystal violet primary staining method, as previously described [12]. First, in 96-well flat-bottom microtiter plates, 20 µL of bacterial log phase culture was added to 180 µL supplemented with 1% glucose trypticase soy broth (TSB, QingdaoHopeBio Technology Co., Ltd., Qingdao, China). After incubation for 24 h at 37 °C under aerobic conditions, the wells were washed three times with 200 µL of sterile phosphate-buffered saline (PBS, pH 7.2), and they were drained by inversion. Subsequently, 200 µL of 95% ethanol was added to every well, and the plates were dried for 30 min. To all plates, 200 µL 0.1% crystal violet solution was added for staining for 15 min, and then the plates were washed with PBS. Finally, crystal violet was dissolved for 15 min using 200 µL of 33% acetic acid, then biofilm formation was measured at 570 nm optical density (OD) for stained bacteria and control wells. This experiment was performed in triplicate. As a negative control, 200 µL TSB + 1% glucose medium was used to determine the background OD [cut-off value (ODc) = average OD of negative control + 3× standard deviation (SD) of negative control] [28]. The quantitative classification of biofilm production, based on ODc and average OD values, was carried out: strong biofilm producers (OD > 4 × ODc), moderate biofilm producers (2 × ODc < OD ≤ 4 × ODc), weak biofilm producers (ODc < OD ≤ 2 × ODc) and non-biofilm producers (OD ≤ ODc), respectively.

### 2.5. Comparative Genomic Analysis of Prevalent Sequence Types of Cronobacter spp. Isolates

To clarify the genetic relatedness of prevalent STs in this study and their global isolates, published WGS datasets containing the same STs were downloaded from the GenBank database and included for comparative genomic analysis (Supplementary Table S2). A total of 179 *Cronobacter* spp. isolates in this study were used to generate a large data matrix to infer phylogenetic relationships. A Mash phylogenetic tree was constructed, based on global mutation distances of the whole genome, using Mash (v2.1) and visualized using iTOL (https://itol.embl.de/, accessed on 22 July 2023).

### 2.6. Statistical Analyses

Data analyses were performed using GraphPad Prism software (Version 6.1.; GraphPad, San Diego, CA, USA).

## 3. Results

### 3.1. Prevalence and Characteristics of Cronobacter spp.

Forty *Cronobacter* spp. strains, including thirty *C. sakazakii*, eight *C. malonaticus*, and two *C. dublinensis* were obtained from surveillance studies of raw milk, silage, bedding, and feces taken from dairy farms. The total isolation rate of *Cronobacter* spp. in dairy farm was 3.59% (40/1115). The isolation rate for raw milk was 0.56% (4/710) and, for silage, bedding, and feces, the isolation rate was 16.0% (16/100), 9.68% (15/155), and 3.33% (5/150), with maximum counts of 3.4 × 10^2^, 1.6 × 10^3^, 2.2 × 10^3^, and 7.4 × 10^2^ CFU/g(mL), respectively (Table 1).

### 3.2. Multilocus Sequence Typing and O-Serotyping

The results of MLST showed a high diversity of strains, with 40 isolates assigned to 31 STs (Table 2). Sequence type 17 (ST17) was the most prevalent, with four isolates, followed by ST23, ST60, ST64, ST125, ST219, and ST940 with two isolates each. Twenty-four of the 31 STs were unique to only one isolate, and three were novel (ST925, ST939, and ST940). Interestingly, ST17 and ST23 were found in both silage and bedding.

Five O-serotypes were identified for C. sakazakii, with O1 (n = 12) and O2 (n = 12) as the most represented serotypes, followed by serotypes O3 (n = 2), O4 (n = 2), and O7 (n = 1), and one isolate was undefined. Eight C. malonaticus isolates were classified into three serotypes, and O1 was the predominant serotype, accounting for one half (4/8) of all C. malonaticus isolates (Table 2). Each strain of C. dublinensis (n = 2) represented unique serotypes. In addition, our data showed a strong correlation between ST and serotype (Table 2).

### 3.3. Antimicrobial Resistance Phenotypes and Genotypes in Cronobacter spp. Isolates

The results of antimicrobial susceptibility tests and antimicrobial resistance gene analysis are shown in Table 2. All examined isolates were susceptible to seven antibiotics, including ampicillin, tetracycline, ciprofloxacin, clindamycin, sulfamethoxazole, meropenem, and chloramphenicol (Table 2), while 100.0% and 70.0% of strains were resistant to cephalothin and fosfomycin, respectively. Also, 12.5% of strains were intermediate to gentamicin (Table 2). Phenotypic resistance correlated strongly with the presence of known resistance determinants encoding for cephalothin and fosfomycin (Table 2). At the genotypic level, all strains carried β-lactam resistance genes, which confer resistance to cephalosporins. For example, *C. dublinensis* isolates carried the *ampC* gene, and *C. sakazakii* and *C. malonaticus* isolates carried the *blaCSA* and *blaCMA* genes, respectively. The gene *fos*, encoding fosfomycin resistance, was present in 97.5% (39/40) strains. In addition, mutants *blaCSA-1* or *blaCMA-2*, of the *blaCSA* and *blaCMA* genes, were found. However, there was no clear association between sources of *Cronobacter* spp. and antimicrobial susceptibility.

### 3.4. Prevalence and Distribution of Virulence Genes

**Figure 1:**
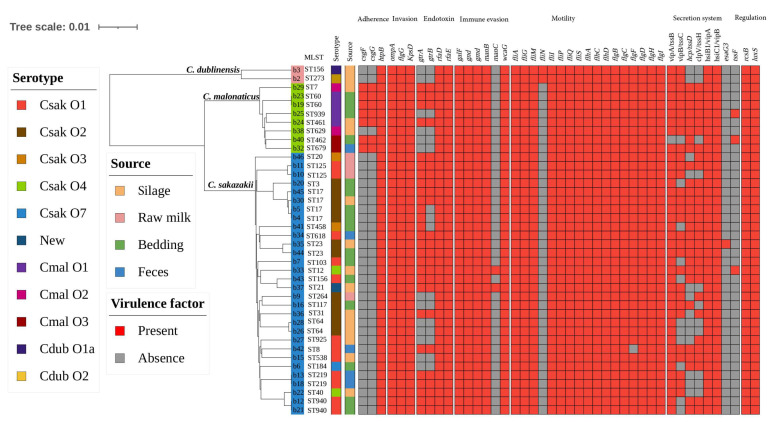
Phylogenetic tree and distribution of virulence genes in dairy farm-associated *Cronobacter* spp. isolates. The phylogenetic tree was constructed based on global mutation distances of the whole genome using Mash and was further visualized using iTOL. Isolation information sources, as well as serotype, are shown with stripes in different colors. The presence and absence of virulence genes are shown with a heat map.

The presence of virulence genes among isolates is shown in Figure 1. Virulence markers present in all isolates included adherence-related gene *htpB*, invasion-related genes *ompA*, *flgG*, and *kpsD*, immune evasion-related genes *gnd*, *galF*, and *manB*, endotoxin-related genes *rfaD* and *rfaE*, regulation-related genes *rcsB* and *luxS*, secretion system-related genes *hsiB1/vipA* and *hsiC1/vipB*, and motility-related genes *flhA*, *flhC*, *flhD*, *flgB*, *flgC*, *flgD*, *flgH*, *flgI*, *fliA*, *fliG*, *fliI*, *fliM*, *fliP*, *fliQ*, and *fliS* (Figure 1). The same virulence genes were detected in *C. sakazakii* and *C. malonaticus* strains, except for the *csgF* and *csgG* genes. In addition, two *C. dublinensis* isolates harbored the same virulence genes, and the motility-related gene *fliN* was only present in *C. dublinensis*.

### 3.5. Biofilm Formation

**Figure 2:**
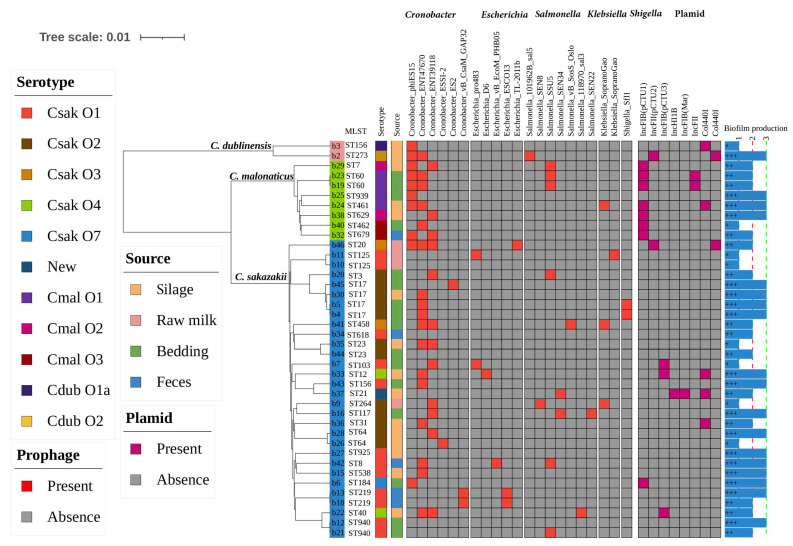
Phylogenetic tree and heat map summary of presence of prophages and plasmids possessed by the 40 *Cronobacter* spp. isolates. The phylogenetic tree was constructed based on global mutation distances of the whole genome using Mash and was further visualized using iTOL. Isolation information sources, as well as serotype, are shown with stripes in different colors. The presence and absence of prophages and plasmids are shown with a heat map. The biofilm production of all 40 isolates were shown with symbols (+ ∼ + + +) and a simple bar chart exhibited their capacity for biofilm formation.

The biofilm formation abilities of 40 *Cronobacter* isolates with 31 STs were tested. Each tested isolate exhibited the capacity for biofilm formation; 42.5% (17/40) of them were capable of forming a strong biofilm, while 37.5% (15/40) formed moderate biofilm, and 20.0% (8/40) formed weak biofilm (Figure 2). The *C. malonaticus* isolates were able to form a strong or moderate type of biofilm (Figure 2).

### 3.6. Presence of Prophages and Other Mobile Genetic Elements

**Figure 3:**
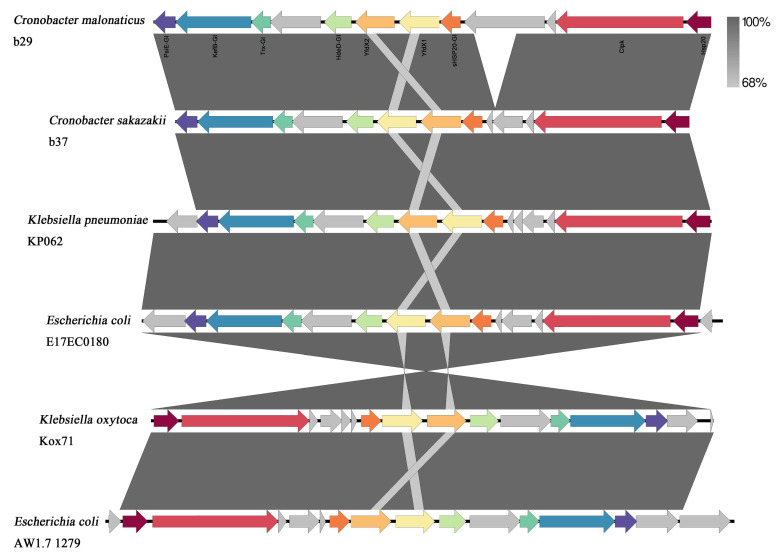
Sequences of transmissible locus of stress tolerance (tLST), including two isolates from this study (b29 and b37), together with an additional four reference sequences sharing high BLAST similarity scores to one of the two (*Escherichia coli* AW1.7 1279, *Escherichia coli* E17EC0180, *Klebsiella pneumoniae* KP062, and *Klebsiella oxytoca* Kox71, respectively).

Prophages were identified in all *Cronobacter* isolates, including five prophages against *Cronobacter*, *Escherichia coli*, *Salmonella*, *Klebsiella pneumoniae*, and *Shigella* (Figure 2). In total, 21 types of prophages were embedded in the *Cronobacter* genome, the top three of which *Cronobacter*_ENT47670 (42.5%, 17/40), *Cronobacter*_ENT39118 (30.0%, 12/40), and *Cronobacter*_phiES15 (25.0%, 10/40) (Figure 2). Notably, we found that one *C. sakazakii* genome carried all three dominant prophages, and 11 isolates of *Cronobacter* spp. contained more than three types of prophages. Moreover, a total of eight plasmids were found in 40 strains of *Cronobacter* spp. The plasmid IncFIB (pCTU1) was the most common one found in *Cronobacter* isolates and was mainly distributed in *C. malonaticus* (7/8). In addition, *C. sakazakii* b29 and *C. malonaticus* b37 contained the tLST, consisting of the small heat shock protein sHSP20 and the heat resistance proteins PsiE-GI, YfdX1, YfdX2, trxLHR, and kefB-GI. The tLST in this study showed high nucleotide similarity to those previously described for other *Enterobacteriaceae* (Figure 3). The genomes of tLST also showed 99.38% nucleotide identity between *C. sakazakii* b29 and *C. malonaticus* b37 isolates (Figure 3).

### 3.7. Minimum Spanning Tree Analysis of Prevalent Sequence Types among Different Sources and between Countries

**Figure 4:**
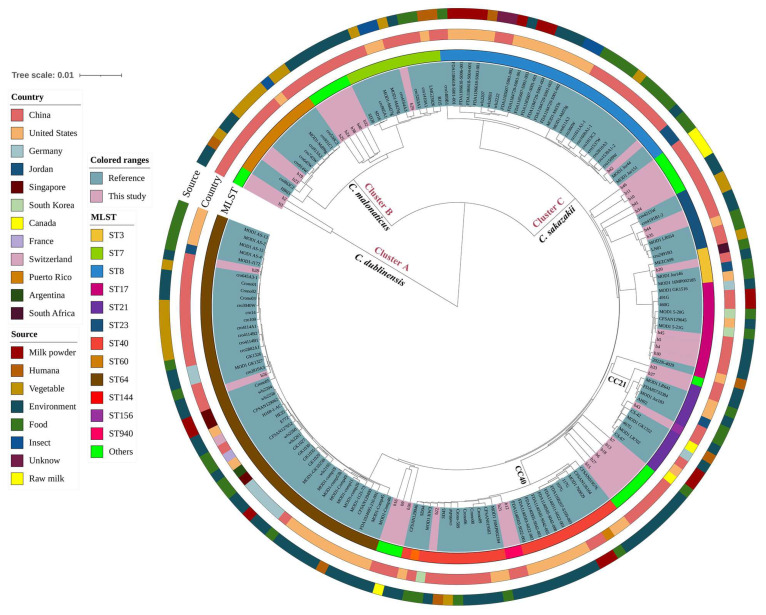
Whole-genome maximum likelihood phylogenetic trees of all 40 *Cronobacter* spp. genomes in this study, combined with 139 reference genomes sharing the same sequence types from NCBI. Strains from this study and publicly available reference genomes are indicated by leaf color. The isolation information of multi-locus sequence typing (MLST), countries, and sources were also color-coded in the following rings. The Mash phylogenetic tree was constructed based on global mutation distances of the whole genome using Mash (v2.1) and cleaned and overlayed using iTOL.

Exploring the evolution of *Cronobacter* spp. isolates, a phylogenetic tree was constructed with our *Cronobacter* isolates and an additional 139 available reference genomes from the NCBI database, including those isolates from outside China that shared the same STs identified in this study (Figure 4). The phylogenetic analysis, based on WGS datasets, revealed that all isolates could be divided into three major clusters to support the species-level divergence patterns, and the same ST strains into smaller evolutionary units, showing more powerful discrimination. There was no direct correlation observed between the STs and the sources or geographical location. Cluster B comprised 27 *C. malonaticus* strains isolated from China and the United States. Cluster C comprised 150 *C. sakazakii* strains, which were mainly isolated from food and environmental samples and varied between countries. Isolate b22 from this study showed a very close relationship with human clinical isolate SD45 (RPCB00000000) from China.

## 4. Discussion

*Cronobacter* has attracted global attention due to its links to neonatal diseases via contaminated powdered infant formula [29]. Raw milk is an important vehicle for this pathogen, although *Cronobacter* spp. are inactivated by pasteurization; nevertheless, this is the raw material for powdered infant formula manufacturing, and it is therefore essential to pay attention to the contamination situation of *Cronobacter* spp. in the dairy farm system. Previous studies have reported the prevalence of *Cronobacter* in powdered infant milk, vegetables, fruits, and plant-derived foods [30]. Farm-associated niches represent a key cross-contamination route for *Cronobacter* spp. in raw milk. So, it is advisable to track and monitor *Cronobacter* spp. in milking and herd hygiene.

In this study, we carried out whole-genome sequencing to investigate the prevalence, genetic phylogeny, and virulence factors of *Cronobacter* spp. from raw milk and its environment in two provinces in China. Forty *Cronobacter* spp. isolates were identified: *C. sakazakii* (n = 30) was detected in both environmental samples and raw milk samples, while *C. malonaticus* (n = 8) and *C. dublinensis* (n = 2) were detected only in environmental samples.

The prevalence of *Cronobacter* spp. in the dairy farm environment was 8.89% (36/405), which is similar to a study from the Czech Republic where the isolation rate of *Cronobacter* spp. was 8.0% (45/445) in plant-based food-related environments [22]. The prevalence of *Cronobacter* spp. in environmental samples (3.33~16.0%) was higher than raw milk samples (0.56%), indicating that there was a key cross-contamination route of *Cronobacter* spp. for raw milk. The presence of *C. sakazakii* in raw milk samples could represent a high risk of contamination of powdered infant formula. As reported in previous studies, the occurrence of *Cronobacter* spp. was related to the environments of farms, and *Cronobacter* spp. can be spread through contaminated lactose powder, soil, and feces [6,12]. Additionally, *Cronobacter* spp. is a plant-associated bacterial microorganism that has been already widely isolated from cereals and derived products of plant origin, such as rice, wheat, oats, and cereals made of corn [31,32,33]. Overall, silage and bedding mainly consist of cereals and their straws; this may be the reason why the detection rates were high in silage and bedding samples. Indeed, the presence of *Cronobacter* spp. is unsurprising in dairy farm plant origin samples, because the plant environment represents a natural habitat for these bacteria. However, incidences in raw milk are bad; it reflects the presence of *Cronobacter* spp. cross-contamination on dairy farms. Therefore, it is necessary to strengthen hygiene management measures to avoid *Cronobacter* spp. cross-contamination spread to raw milk.

MLST indicates that *Cronobacter* spp. is highly genetically diverse, since the 40 *Cronobacter* spp. strains were assigned to 31 STs, which is a ratio of 1.3 strains to each ST found. Li et al. tested STs of *Cronobacter* spp. from powdered infant formula and processing environments, and a total of 35 STs were obtained in 35 *Cronobacter* spp. isolates [34]. Pathogenic STs (ST3, ST7, ST8, ST17, ST23, ST40, ST60, and ST64) associated with clinical infections were also observed. Many studies have shown that *C. sakazakii* ST64 is one of the most prevalent sequence types in powdered infant formula and processing environments in China [34,35]. One case of *C. malonaticus* ST60 in an infected infant was reported in Wuhan, in the Hubei Province of China [36]. *C. malonaticus* ST7 has been isolated in different age groups, from infants to adults [37]. The frequent detection of global pandemic-relevant STs in dairy farms highlights that it is necessary to ongoing surveillance of these STs.

Isolates belonging to ST925, ST939, and ST940 were first described in this study and were unique in the database. Additionally, ST156 belonged to clonal complex 21 (CC21), which was a double-locus variant of ST21. Novel sequence type ST940 belonged to CC40, identified as a single-locus variant of ST40. A total of eleven *Cronobacter* serogroups were recognized in this study. *C. sakazakii* O1 and O2 were the dominant serotypes, in accordance with a previous study from China which investigated commercial powdered infant formula [8]. Additionally, another previous study showed that *C. sakazakii* serotypes O1, O2, and O4, and *C. malonaticus* serotype O2 were particularly significant in clinical cases and were probably related to human infections [38]. Of these serotypes, 70% (28/40) in this study may be a potential risk to food safety and consumer health.

The antimicrobial resistance rate of *Cronobacter* spp. isolated in this study was found to be low overall, similar to previous studies [39]. It is worth noting that *Cronobacter* spp. was highly resistant to cephalothin (100%) and fosfomycin (70.0%), and carried relevant antimicrobial resistance known genotypes. The resistance of *Cronobacter* spp. to cephalothin and relevant resistance genotypes (*blaCSA* and *blaCMA*) has been reported in several previous studies [17,40]. Previous studies rarely reported *Cronobacter* spp. that were resistant to fosfomycin; 97.5% (39/40) of strains carried the gene *fos*, which is responsible for fosfomycin resistance, in this study. A recent study from infant supplement food in China found two *C. sakazakii* strains showed resistance to ampicillin, tetracycline, sulfamethoxazole–trimethoprim, and chloramphenicol [41]. Another recent report on powdered infant formula milk in Iran showed that 96% (24/25) of *C. sakazakii* isolates were multi-drug resistant (to at least three different antibiotic classes), and that eight isolates were resistant to a profile consisting of six different classes of antibiotics [17]. Additionally, a plasmid-borne colistin resistance gene *mcr-9.1* was found in *C. sakazakii* [18]. Therefore, we need to use these antimicrobial agents with caution when managing *Cronobacter* spp.

We still do not have a complete understanding of how *Cronobacter* causes disease, because its pathogenicity mechanisms are complex. A recent study by Jang at al. reported some common virulence factors shared among seven *Cronobacter* species and described the exoproteins of toxins secreted by *Cronobacter* [42]. In the present study, virulence factors were evaluated based on WGS. Virulence factors were grouped into adherence, invasion, immune evasion, endotoxin, regulation, secretion system, and motility. The virulence factors associated with invasion (*ompA*, *flgG*, and *kpsD*) and regulation (*rcsB* and *luxS*) were detected in all genomes of *Cronobacter* spp. It has been confirmed elsewhere that the *ompA* gene plays a crucial role in the invasion of host cells by *C. sakazakii* [43]. Flagella are primarily responsible for bacterial motility, and motility-related genes mainly encode flagella proteins. Holý et al. found that the motility-related gene *fliC* was detected in all *Cronobacter* spp. isolates, whereas genes *fliA*, *fliG*, and *fliM* were detected in this study [44]. None of the *C. sakazakii* and *C. malonaticus* strains harbored the *fliN* gene, but it was present in both of the *C. dublinensis* strains. This may be considered a distinctive trait of *C. dublinensis*. The type VI secretion system is the most common secretory system in *Cronobacter* spp., and it is involved in virulence, host immunity resistance, and interbacterial interaction [45]. All of the secretion system-related genes were type VI in this study, which is similar to a report by Wang et al., who reported that all 138 *C. sakazakii* strains possessed a type VI secretion system [46].

Prophages are a mobilizable segment of the bacterial genome and one of the vehicles for the horizontal transfer of virulence factors, which is crucial for driving the evolution of new virulent lineages of bacteria. Prophages (ENT47670, ENT39118, and phiES15) were the most prevalent in this study, and complete genomes of them were reported in 2012 [47,48]. Interestingly, prophages of *Escherichia coli*, *Salmonella*, *Klebsiella pneumoniae*, and *Shigella* were also detected in the *Cronobacter* genomes. Jang et al., using the online analysis tool PHASTER, analyzed prophages of 88 *C. sakazakii* strains originating from plant-origin foods, and a total of eight different bacterial species prophages were detected [49]. Although there are no virulence genes associated with these prophages, it is well known that prophages are an important feature for bacterial competition and genetic diversity. Possessing multiple prophages and prophage-related genes may increase the survival and pathogenicity of *Cronobacter* strains.

Stress tolerance bacteria are a serious food safety and public health concern. Several studies have reported the ability of *Cronobacter* spp. to produce biofilms [50]. A greater capacity for biofilm formation by bacteria suggests a greater capacity for environmental adaptability. This is an important risk factor for persistent contamination in food samples and food processing environments. The tLST is a genomic island that confers stress resistance and has been discovered in different species of *Enterobacteriaceae*. The prevalence rates of tLST in *Escherichia coli*, *Salmonella*, *Klebsiella pneumoniae*, and *Cronobacter* are, approximately, 2%, 0.1%, 3%, and 8%, respectively [51]. *Cronobacter* spp. strains harboring the tLST showed a stronger survival ability in four temperatures (56, 58, 60, and 62 °C) after heat shock (53 °C for 15 min) [52]. Two strains harboring the tLST were found in this study, which may lead to their increased survival during the milk powder manufacturing process, due to their greatly elevated heat resistance.

Whole-genome maximum likelihood phylogenetic trees, including the 40 genomes in this study and an additional 139 international reference genomes, revealed that all isolates could divided into three major species-level clusters, and the same STs of *Cronobacter* isolates formed their own smaller clusters. Close genetic relatedness was found in the phylogenetic tree between environmental, food, vegetable, milk powder, clinical, and outbreak strains. Furthermore, almost half (45.81%) of *Cronobacter* spp. isolates from environments observed in global epidemiological investigations suggested the cross-contamination of *Cronobacter* spp. However, this conclusion needed more experimental evidence and more extensive sampling to verify. Overall, our data corroborated that there is a genetic relationship between isolates from various sources on the dairy farm, implying the spatial association and transmission of *Cronobacter* spp. strains.

## 5. Conclusions

In this study, we investigated the prevalence rate, antimicrobial resistance, biofilm formation, and genetic diversity of *Cronobacter* spp. strains isolated from dairy farms in Jiangsu and Shandong provinces in China. MLST and O-Serotyping analyses indicate that *Cronobacter* spp. is highly diverse in these environments. We identified that all isolates were highly resistant to cephalothin and fosfomycin, by antimicrobial phenotype and genotype, and that two strains harbored the transmissible locus of stress tolerance (tLST). In addition, clinically important serotypes and pathogenic STs of *Cronobacter* strains were detected in this study. Thus, the continuous dynamic monitoring of *Cronobacter* spp. on dairy farms is necessary, due to the risk associated with the contamination of powdered infant formula by *Cronobacter* spp.

## Figures and Tables

**Table 1:** Prevalence and contamination level of *Cronobacter* spp. in dairy farms.

Sample	No. of Samples	No. (%) of Positive Samples	*Cronobacter* spp. Counts	
			Minimum	Maximum
raw milk	710	4 (0.56%)	3.1 × 10	3.4 × 10^2^
silage	100	16 (16.0%)	4.4 × 10	1.6 × 10^3^
bedding	155	15 (9.68%)	4.3 × 10	2.2 × 10^3^
feces	150	5 (3.33%)	2.7 × 10	7.4 × 10^2^

**Table 2:** Molecular characteristics, antimicrobial resistance phenotypes, and genotypes of *Cronobacter* spp. isolates ^1^.

Strains	Species	Source	MLST	Serotype	Antibiotics Sensitivity ^2^	Antimicrobial Resistance Gene
b2	*C. dublinensis*	silage	273	Cdub O2	R(CEP)	*ampC*, *fos*
b3	*C. dublinensis*	silage	561	Cdub O1a	R(CEP)	*ampC*, *fos*
b24	*C. malonaticus*	silage	461	Cmal O1	R(CEP)R(FOS)	*blaCMA*, *fos*
b29	*C. malonaticus*	silage	7	Cmal O2	R(CEP)	*blaCMA*, *fos*
b38	*C. malonaticus*	silage	629	Cmal O2	R(CEP)R(FOS)	*blaCMA*, *fos*
b19	*C. malonaticus*	bedding	60	Cmal O1	R(CEP)R(FOS)	*blaCMA*, *fos*
b23	*C. malonaticus*	bedding	60	Cmal O1	R(CEP)	*blaCMA*, *fos*
b25	*C. malonaticus*	bedding	939	Cmal O1	R(CEP)R(FOS)	*blaCMA*
b40	*C. malonaticus*	bedding	462	Cmal O3	R(CEP)R(FOS)	*blaCMA*-*2*, *fosA*
b32	*C. malonaticus*	feces	679	Cmal O3	R(CEP)R(FOS)	*blaCMA*, *fos*
b9	*C. sakazakii*	raw milk	264	Csak O2	R(CEP)R(FOS)	*blaCSA*, *fos*
b10	*C. sakazakii*	raw milk	125	Csak O1	R(CEP)R(FOS)	*blaCSA*, *fos*
b11	*C. sakazakii*	raw milk	125	Csak O1	R(CEP)R(FOS)	*blaCSA*, *fos*
b46	*C. sakazakii*	raw milk	20	Csak O3	R(CEP)R(FOS)I(GEN)	*blaCSA*, *fos*
b26	*C. sakazakii*	silage	64	Csak O2	R(CEP)R(FOS)	*blaCSA*, *fos*
b37	*C. sakazakii*	silage	21	new	R(CEP)R(FOS)	*blaCSA*, *fos*
b27	*C. sakazakii*	silage	925	Csak O1	R(CEP)R(FOS)	*blaCSA*, *fos*
b15	*C. sakazakii*	silage	538	Csak O1	R(CEP)	*blaCSA*, *fos*
b28	*C. sakazakii*	silage	64	Csak O2	R(CEP)	*blaCSA*, *fos*
b22	*C. sakazakii*	silage	40	Csak O4	R(CEP)	*blaCSA*, *fos*
b36	*C. sakazakii*	silage	31	Csak O2	R(CEP)R(FOS)	*blaCSA*, *fos*
b35	*C. sakazakii*	silage	23	Csak O2	R(CEP)R(FOS)	*blaCSA*, *fos*
b30	*C. sakazakii*	silage	17	Csak O2	R(CEP)R(FOS)	*blaCSA*, *fos*
b33	*C. sakazakii*	silage	12	Csak O4	R(CEP)R(FOS)	*blaCSA*, *fos*
b20	*C. sakazakii*	bedding	3	Csak O2	R(CEP)R(FOS)	*blaCSA*, *fos*
b4	*C. sakazakii*	bedding	17	Csak O2	R(CEP)	*blaCSA*, *fos*
b5	*C. sakazakii*	bedding	17	Csak O2	R(CEP)	*blaCSA*, *fos*
b21	*C. sakazakii*	bedding	940	Csak O1	R(CEP)	*blaCSA*, *fos*
b12	*C. sakazakii*	bedding	940	Csak O1	R(CEP)R(FOS)	*blaCSA*, *fos*
b41	*C. sakazakii*	bedding	458	Csak O3	R(CEP)R(FOS)	*blaCSA*, *fos*
b6	*C. sakazakii*	bedding	184	Csak O7	R(CEP)	*blaCSA*, *fos*
b43	*C. sakazakii*	bedding	156	Csak O1	R(CEP)R(FOS)I(GEN)	*blaCSA*, *fos*
b16	*C. sakazakii*	bedding	117	Csak O2	R(CEP)	*blaCSA*, *fos*
b7	*C. sakazakii*	bedding	103	Csak O1	R(CEP)R(FOS)	*blaCSA*, *fos*
b44	*C. sakazakii*	bedding	23	Csak O2	R(CEP)R(FOS)I(GEN)	*blaCSA*, *fos*
b45	*C. sakazakii*	bedding	17	Csak O2	R(CEP)R(FOS)I(GEN)	*blaCSA*, *fos*
b34	*C. sakazakii*	feces	618	Csak O1	R(CEP)R(FOS)	*blaCSA*, *fos*
b18	*C. sakazakii*	feces	219	Csak O1	R(CEP)R(FOS)	*blaCSA*, *fos*
b13	*C. sakazakii*	feces	219	Csak O1	R(CEP)R(FOS)	*blaCSA*, *fos*
b42	*C. sakazakii*	feces	8	Csak O1	R(CEP)R(FOS)I(GEN)	*blaCSA*-*1*, *fos*

^1^: CEP = cephalothin; FOS = fosfomycin; GEN = gentamicin. ^2^: R = resistance; I = intermediary.

## Data Availability

The original contributions presented in the study are included in the article/Supplementary Materials; further inquiries can be directed to the corresponding authors.

## Supplementary Materials

The following supporting information can be downloaded at https://www.mdpi.com/article/10.3390/foods13060871/s1. Table S1: Metadata, the specific information of all samples. Table S2: Information of 139 public *Cronobacter* strains used in this study.
